# Assessing the Utility of the Metabolic Score for Insulin Resistance (METS-IR) in Evaluating Metabolic Risk Among Individuals Undergoing Master Health Checkups in a Tertiary Care Hospital in South India: A Retrospective Cohort Study

**DOI:** 10.7759/cureus.70289

**Published:** 2024-09-26

**Authors:** Mohammed Suhail Tazeem, Nirmala Devi Chandrasekaran, Niveda Srivatsa

**Affiliations:** 1 General Medicine, Sri Ramaswamy Memorial (SRM) Medical College Hospital and Research Centre, SRM Institute of Science and Technology (SRMIST), Chengalpattu, IND; 2 Geriatrics, Sri Ramaswamy Memorial (SRM) Medical College Hospital and Research Centre, SRM Institute of Science and Technology (SRMIST), Chengalpattu, IND

**Keywords:** insulin resistance, master health checkup, metabolic score for insulin resistance (mets-ir), metabolic syndrome and endocrinology, south indian

## Abstract

Background

The metabolic score for insulin resistance (METS-IR) is a neoteric score for assessing insulin resistance that has been used as a non-insulin-based, objectively measured method. It is an easily accessible tool that can be used on a large scale to detect insulin resistance in a community.

Methods

We conducted a retrospective cohort study to explore the utility of this score in identifying metabolic risk in those individuals attending a master health checkup in a tertiary care setting. Data were collected from 254 individuals between October and December 2023.

Results

According to the univariate regression analysis, METS-IR had a strong correlation in predicting cardiovascular health risks, as evidenced by its positive linear association with an increase in age (β=0.186, *p*=0.003), weight (β=0.534, *p*<0.001), waist circumference (β=0.405, *p*<0.001), and body mass index (BMI; β=0.635, *p*<0.001). This explained the value of this score in depicting adiposity and insulin resistance. Lab parameters that showed a significant association were fasting blood sugar (β=0.176, *p*=0.005) and fasting triglycerides (β=0.175, *p*=0.005).

According to the multivariate regression analysis, METS-IR had a significant positive association with fasting blood sugar (*B*=0.489, *p*<0.001) and fasting triglycerides (*B*=0.022, *p*=0.003), implicating its importance in cardiovascular health.

High-density lipoprotein cholesterol (HDL-c; *B*=-0.168, *p*=0.005) confirmed its protective role with its negative association in higher quartile groups. An increase in serum albumin levels (*B*=-0.168, *p*=0.005) and raised gamma-glutamyl transpeptidase (GGT) (*B*=0.059, *p*=0.022) portrays its due diligence in liver health.

METS-IR had a weak association with the estimated glomerular filtration rate (eGFR) with Pearson's correlation coefficient of 0.020 (*p*=0.756) and Spearman's rho of 0.021 (*p*=0.739). However, raised serum creatinine had a significant association in higher quartile groups, with a *p-*value of 0.018.

Conclusions

METS-IR is useful as a screening tool for predicting cardiovascular disease. However, the complex interplay of other confounding factors in identifying renal dysfunction has yet to be explored when considering this score in our study population.

## Introduction

Metabolic syndrome and insulin resistance are central components in the development and progression of chronic conditions that affect millions of individuals globally [1]. These metabolic abnormalities contribute to a range of complications, including type 2 diabetes mellitus, cardiovascular disorders, chronic kidney disease (CKD), and metabolic dysfunction-associated steatotic liver disease (MASLD) [2,3]. Understanding the early metabolic changes and their impact is essential for the effective prevention and management of the ensuing complications.

Insulin resistance is characterized by reduced cellular responsiveness to insulin, leading to impaired glucose uptake and metabolism, as much as it does in a normal population [4]. Over the years, methods have been developed to measure insulin resistance, such as the Homeostatic Model Assessment of Insulin Resistance (HOMA-IR) and the euglycemic clamp technique [5]. Although HOMA-IR is widely used for its simplicity and cost-effectiveness, the euglycemic clamp remains the gold standard. Due to their complexity and invasiveness, the metabolic score for insulin resistance (METS-IR) score was developed, a novel and more promising non-invasive tool for screening insulin sensitivity.

The METS-IR is a recently validated score that measures insulin sensitivity against the hyperinsulinemic-euglycemic clamp (HEC). It is non-insulin-based [6] and calculated using biochemical parameters including fasting glucose, triglycerides (TG), and HDL cholesterol. The METS-IR score provides a practical and accessible alternative to more complex methods. However, the utility of this score has not been extensively explored in Indian populations.

This retrospective cohort study aimed to investigate the utility of the METS-IR score in identifying metabolic and renal health risks and other risks in individuals undergoing a master health checkup at a tertiary care hospital in South India. By examining individual parameters of the METS-IR score and exploring trends in estimated glomerular filtration rate (eGFR) and METS-IR across health disorders and conditions, this study seeks to provide valuable and useful insights into the complex interplay among insulin resistance, metabolic syndrome, and kidney health.

## Materials and methods

Study design and participant selection

This retrospective cohort study analyzed the data of 254 individuals collected from October to December 2023 from a master health checkup clinic at a tertiary healthcare center in South India. All individuals aged ≥20 years who attended the master health checkup with complete records for fasting glucose, lipid profile, and other metabolic parameters were included. Individuals with incomplete records, known end-stage renal disease, and pregnant women were excluded.

Data collection

Data were meticulously extracted from electronic medical records by the study's authors, ensuring accuracy and completeness. The parameters collected included demographic information such as age, gender, smoking status (current, past, never), alcohol consumption (yes/no, frequency), and exercise activity (frequency, type, duration). Anthropometric measurements such as height in cm, weight in kg, waist circumference (WC) in cm, and body mass index (BMI, kg/m^2^) were collected. Biochemical parameter assessments were done, which included fasting blood sugar (FBS, mg/dL), postprandial blood sugar (PPBS, mg/dL), glycated hemoglobin (HbA1c, %), total cholesterol (TC, mg/dL), low-density lipoprotein cholesterol (LDL-c, mg/dL), high-density lipoprotein cholesterol (HDL-c, mg/dL), aspartate aminotransferase (AST, IU/L), alanine aminotransferase (ALT, IU/L), gamma-glutamyl transferase (GGT, IU/L), blood urea nitrogen (BUN, mg/dL), urea (mg/dL), serum creatinine (mg/dL), albumin (g/dL), globulin (g/dL), and total protein (g/dL).

The METS-IR score was calculated to provide insights into insulin resistance and metabolic syndrome. Renal functions tests were done, eGFR (mL/min/1.73 m^2^), and the classification of CKD stages was based on CKD-EPI criteria. Liver health was assessed through ultrasonography (USG) grading of fatty liver using a convex curved array probe at a frequency of approximately 2-3 MHz. Diabetic status was categorized into no diabetes, incident prediabetes, incident diabetes, and established diabetes, each defined as: no diabetes (FBS<100 mg/dL and HbA1c<5.7%), incident prediabetes (FBS between 100 and 125 mg/dL or HbA1c between 5.7 and 6.4%, with no prior history of prediabetes or diabetes), incident diabetes (FBS≥126 mg/dL or HbA1c≥6.5%, newly diagnosed during the study period), and established diabetes (defined as having a documented history of diabetes before the study period).

The METS-IR score was calculated using the formula: Ln ((2 x fasting glucose (mg/dL)) + fasting TG (mg/dL)) x BMI (kg/m^2^)) / (Ln (HDL-c (mg/dL)) [6]. The eGFR was estimated using the CKD-EPI creatinine equation 2021 [7,8]. CKD stages were classified based on eGFR values using the CKD-EPI classification [9].

Ultrasonography reports were reviewed to grade fatty liver disease as Grade 0 (no fatty liver), Grade 1 (mild steatosis/echogenicity was only marginally increased), Grade 2 (moderate steatosis/when the echogenic liver obscured the echogenic walls of portal vein branches), and Grade 3 (severe steatosis/echogenic liver obscured the diaphragmatic outline) [10].

Ethical considerations

This study has received ethical clearance from the Institutional Ethics Committee of SRM Medical College Hospital & Research Centre (SRMIEC-ST0823-579). All patient data were anonymized to ensure confidentiality and privacy.

Statistical analysis

Data analysis was conducted using IBM SPSS Statistics for Windows, Version 27 (Released 2020; IBM Corp., Armonk, New York), with statistical significance set at p<0.05. Descriptive statistics such as mean, median, standard deviation, and frequencies were utilized to summarize demographic and clinical characteristics across the different quartiles based on the distribution of METS-IR scores. ANOVA was used for normally distributed continuous variables, and the Kruskal-Wallis test was employed for non-normally distributed data. Chi-square tests were employed to compare proportions across different categorical variables across the quartiles. Pearson's correlation coefficient was used to assess the linear relationship between METS-IR scores and eGFR for normally distributed data, and Spearman's correlation coefficient was used for non-normally distributed data. Multivariate linear regression models were constructed to determine the independent association between the METS-IR scores and eGFR.

## Results

The METS-IR scores in each quartile were as follows: Q1 scores below 35.25, Q2 scores from 35.250 to 40.7, Q3 scores from 40.700 to 44.9, and Q4 scores above 44.900.

A detailed comparison of baseline characteristics across four quartiles of METS-IR is shown in Table 1, which revealed significant associations with clinical parameters. The mean age increased significantly from Q1 to Q4 (p=0.036), suggesting an association between older age and higher METS-IR. Significant increases in weight, WC, and BMI across the quartiles (p<0.001) indicated that higher adiposity is linked to higher METS-IR. Blood glucose levels, including FBS (p=0.046) and PPBS (p=0.036), as well as HbA1c levels (p=0.010), increased significantly, highlighting poorer glycemic control with higher METS-IR. Dyslipidemia is evidenced by rising fasting TG (p=0.002) and LDL-c levels (p=0.009). Although eGFR showed some variation, it was not significantly different across the quartiles (p=0.062). Significant differences in CKD stages (p=0.007), BUN (p=0.023), and serum creatinine levels (p=0.018) suggested renal function variation with METS-IR. No significant differences were observed in gender distribution, smoking status, alcohol consumption, exercise activity, or diabetic status across the quartiles.

**Table 1:** Baseline characteristics against METS-IR BMI, body mass index; FBS, fasting plasma glucose; PPBS, post-prandial plasma glucose; METS-IR, metabolic score for insulin resistance; eGFR, estimated glomerular filtration rate; TG, triglycerides; LDL-C, low-density lipoprotein cholesterol; HDL-C, high‐density lipoprotein cholesterol; AST, aspartate aminotransferase; ALT, alanine aminotransferase; GGT, gamma-glutamyl transpeptidase; BUN, blood urea nitrogen; CKD, chronic kidney disease; USG, ultrasonography; Q1, Q2, Q3, and Q4 are quartiles of the metabolic score for insulin resistance(METS-IR); SD, standard deviation; IQR, interquartile range.

Parameter	Total (N=254) Mean ± SD	Q1 (N=63) Mean ± SD	Q2 (N=65) Mean ± SD	Q3 (N=63) Mean ± SD	Q4 (N=63) Mean ± SD	Median	IQR	P-value (ANOVA)	Kruskal-Wallis H-test
Age	45.13 ± 15.24	42.57 ± 19.9	44.5 ± 14.5	43.95 ± 12.86	49.46 ± 13.75	43	25	0.061	0.036
Height (cm)	164.18 ± 7.02	163.97 ± 6.61	164.35 ± 7.35	164.9 ± 6.99	163.49 ± 7.19	165.0	7.25	0.714	0.706
Weight (kg)	69.09 ± 12.56	55.49 ± 8.41	66.09 ± 8.34	74.16 ± 6.99	80.73 ± 9.53	69.0	17.33	<0.001	<0.001
Waist circumference (cm)	92.38 ± 18.55	77.81 ± 12.96	86.76 ± 11.94	97.41 ± 16.35	107.67 ± 17.62	90.0	22.50	<0.001	<0.001
BMI (kg/m^2^)	25.64 ± 4.25	20.65 ± 2.35	24.44 ± 2.33	27.25 ± 1.8	30.25 ± 2.87	26.0	5.6	<0.001	<0.001
FBS (mg/dL)	122.25 ± 63.42	109.08 ± 54.26	124.66 ± 71.67	115.94 ± 51.74	139.24 ± 70.53	97.50	41	0.046	0.010
PPBS (mg/dL)	175.14 ± 99.97	156.06 ± 83.1	180.23 ± 115.06	163 ± 91.45	201.1 ± 103.1	134.50	116	0.053	0.036
HbA1c (%)	6.73 ± 2.19	6.12 ± 1.46	7.15 ± 2.76	6.41 ± 1.86	7.21 ± 2.3	6.0	1.7	0.008	0.010
METS-IR	40.92 ± 9.82	31.06 ± 3.74	38.26 ± 1.57	42.71 ± 1.25	51.74 ± 12.12	40.7	9.6	<0.001	<0.001
eGFR (mL/min/1.73 m^2^)	99.98 ± 22.32	95.29 ± 25.53	100.72 ± 24.27	105.21 ± 20.38	98.7 ± 17.45	103.50	29	0.088	0.062
Fasting TG (mg/dL)	127.34 ± 73.77	106.79 ± 54.17	122.55 ± 70.66	131.95 ± 46.96	148.21 ± 104.85	111.5	65	0.014	0.002
Total cholesterol (mg/dL)	185 ± 44.1	178.22 ± 44.27	186.57 ± 42.69	191.9 ± 39.15	183.27 ± 49.61	183.5	59	0.362	0.306
LDL-c (mg/dL)	123.27 ± 37.85	111.68 ± 44.46	125.88 ± 34.37	131.57 ± 32.13	123.87 ± 37.45	123.0	47	0.025	0.009
HDL-c (mg/dL)	42.3 ± 10.02	44.49 ± 13.11	41.98 ± 8.82	42.59 ± 8.28	40.13 ± 8.9	41.0	11	0.107	0.254
AST (IU/L)	29.17 ± 44.59	30.76 ± 24.31	25.42 ± 12.37	24.76 ± 10.16	35.84 ± 84.73	23.0	10	0.465	0.172
ALT (IU/L)	28.22 ± 23.7	29.97 ± 29.05	28.02 ± 17.61	26.09 ± 14.97	28.79 ± 29.94	22.0	16	0.829	0.986
GGT (IU/L)	32.18 ± 27.89	34.86 ± 27.97	27.97 ± 15.49	30.75 ± 17.38	35.29 ± 25.09	26	19.25	0.392	0.606
BUN (mg/dL)	10.85 ± 4.85	11.79 ± 10.69	10.69 ± 5.29	9.86 ± 4.34	11.05 ± 5.26	9.0	5	0.157	0.023
Urea (mg/dL)	22.16 ± 9.33	23.32 ± 22.23	22.23 ± 11.16	20.89 ± 8.82	22.21 ± 9.27	20.0	9	0.545	0.130
Serum creatinine (mg/dL)	0.86 ± 0.27	0.943 ± 0.860	0.86 ± 0.31	0.82 ± 0.29	0.82 ± 0.17	0.9	0.3	0.036	0.018
Albumin (g/dL)	4.51 ± 5.71	5.61 ± 4.12	4.1 ± 0.37	4.22 ± 0.3	4.12 ± 0.4	4.2	0.4	0.377	0.084
Globulin (g/dL)	3.11 ± 0.44	3.12 ± 3.05	3.05 ± 0.46	3.17 ± 0.39	3.13 ± 0.43	3.1	0.4	0.487	0.321
Total protein (g/dL)	7.63 ± 5.7	8.73 ± 11.38	7.15 ± 0.58	7.39 ± 0.5	7.25 ± 0.61	7.3	0.7	0.368	0.161
Gender, n (%)	-	-	-	-	-	-	-	0.892	0.893
Male	152 (59.8%)	38 (60.3%)	37 (56.9%)	40 (63.5%)	37 (58.7%)	-	-	-	-
Female	102 (40.2%)	25 (39.7)	28 (43.1%)	23 (36.5%)	26 (48.3%)	-	-	-	-
Smoking status, n (%)	-	-	-	-	-	-	-	0.434	0.436
None or past	165 (65%)	42 (66.7%)	46 (70.8%)	36 (57.1%)	41 (65.1%)	-	-	-	-
Current	89 (35%)	21 (33.3%)	19 (29.2%)	27 (42.9%)	22 (34.9%)	-	-	-	-
Alcohol consumption, n (%)	-	-	-	-	-	-	-	0.221	0.517
None or minimal	129 (50.8%)	27 (42.9%)	36 (55.4%)	31 (49.2%)	35 (55.6%)	-	-	-	-
Light	42 (16.5%)	17 (27%)	11 (16.9%)	6 (9.5%)	8 (12.7%)	-	-	-	-
Moderate	72 (28.3%)	15 (23.8%)	17 (26.2%)	23 (36.5%)	17 (27%)	-	-	-	-
Heavy	11 (4.3%)	4 (6.3%)	1 (1.5%)	3 (4.8%)	3 (4.8%)	-	-	-	-
Exercise activity, n (%)	-	-	-	-	-	-	-	0.987	0.987
No	117 (46.1%)	28 (44.4%)	31 (47.7%)	29 (46%)	29 (46%)	-	-	-	-
Yes	137 (53.9%)	35 (55.6%)	34 (52.3%)	34 (54%)	34 (54%)	-	-	-	-
CKD stage, n (%)	-	-	-	-	-	-	-	0.036	0.007
Stage I	182 (71.7%)	36 (57.1%)	48 (73.8%)	54 (85.7%)	44 (69.8%)	-	-	-	-
Stage II	60 (23.6%)	22 (34.9%)	14 (21.5%)	6 (9.5%)	18 (28.6%)	-	-	-	-
Stage IIIa	8 (3.1%)	3 (4.8%)	2 (3.1%)	2 (3.2%)	1 (1.6%)	-	-	-	-
Stage IIIb	2 (0.8%)	2 (3.2%)	0 (0%)	0 (0%)	0 (0%)	-	-	-	-
Stage IV	2 (0.8%)	0 (0%)	1 (1.5%)	1 (1.6%)	0 (0%)	-	-	-	-
USG fatty liver, n (%)	-	-	-	-	-	-	-	0.221	0.144
Grade 0	148 (58.3%)	45 (71.4%)	36 (55.4%)	34 (54%)	33 (52.4%)	-	-	-	-
Grade 1	77 (30.3%)	12 (19%)	24 (36.9%)	20 (31.7%)	21 (33.3%)	-	-	-	-
Grade 2	29 (11.4%)	6 (9.5%)	5 (7.7%)	9 (14.3%)	9 (14.3%)	-	-	-	-
Diabetic status, n (%)	-	-	-	-	-	-	-	0.130	0.138
No diabetes	90 (35.4%)	28 (44.4%)	25 (38.5%)	23(36.5%)	14 (22.2%)	-	-	-	-
Incident diabetes	25 (9.8%)	5 (7.9%)	4 (6.2%)	5 (7.9%)	11 (17.5%)	-	-	-	-
Prediabetic	71 (28%)	17 (27%)	17 (26.2%)	21 (33.3%)	16 (25.4%)	-	-	-	-
Diabetic	68 (26.8%)	13 (20.6%)	19 (29.2%)	14 (22.2%)	22 (34.9%)	-	-	-	-

Significant associations between METS-IR and several key factors were found by performing univariate regression analysis, shown in Table 2 on the dataset. Age (β=0.186, p=0.003), weight (β=0.534, p<0.001), WC (β=0.405, p<0.001), and BMI (β=0.635, p<0.001) all showed strong positive associations with METS-IR, indicating the critical impact of adiposity on insulin resistance. Fasting blood sugar (β=0.176, p=0.005) and fasting TG (β=0.175, p=0.005) were also significantly associated with METS-IR, underscoring the importance of glycemic control and lipid metabolism. Other variables, such as height, eGFR, total cholesterol, LDL-c, HDL-c, liver enzymes, renal function parameters, other metabolic parameters, gender, smoking status, alcohol consumption, exercise activity, CKD stage, USG fatty liver, and diabetic status, did not show significant associations with METS-IR.

**Table 2:** Results of univariate analysis of METS-IR in the whole population BMI, body mass index; FBS, fasting plasma glucose; PPBS, post-prandial plasma glucose; METS-IR, metabolic score for insulin resistance; eGFR, estimated glomerular filtration rate; TG, triglycerides; LDL-C, low-density lipoprotein cholesterol; HDL-C, high‐density lipoprotein cholesterol; AST, aspartate aminotransferase; ALT, alanine aminotransferase; GGT, gamma-glutamyl transpeptidase; BUN, blood urea nitrogen; CKD, chronic kidney disease; USG, ultrasonography; B, unstandardized coefficient; CI, confidence interval; β, standardized coefficient.

Variable	B (95% CI for B)	β	P-value
Age	0.120(0.041, 0.198)	0.186	0.003
Height (cm)	-0.101(-0.275, 0.072)	-0.072	0.250
Weight (kg)	0.418(0.336, 0.500)	0.534	<0.001
Waist circumference (cm)	0.214(0.154,0.274)	0.405	<0.001
BMI (kg/m^2^)	1.467(1.245, 1.688)	0.635	<0.001
FBS (mg/dL)	0.027(0.008, 0.046)	0.176	0.005
PPBS (mg/dL)	0.011(-0.001, 0.023)	0.110	0.080
HbA1c (%)	0.506(-0.046, 1.058)	0.113	0.072
METS-IR	Dependent variable	-	-
eGFR (mL/min/1.73 m^2^)	0.009(-0.046, 0.063)	0.020	0.756
Fasting TG (mg/dL)	0.023(0.007, 0.040)	0.175	0.005
Total cholesterol (mg/dL)	-0.004(-0.031, 0.024)	-0.017	0.790
LDL-c (mg/dL)	0.010(-0.022, 0.042)	0.037	0.553
HDL-c (mg/dL)	-0.101(-0.222, 0.020)	-0.103	0.102
AST (IU/L)	0.007(-0.020, 0.034)	0.032	0.608
ALT (IU/L)	-0.002(-0.054, 0.049)	-0.005	0.931
GGT (IU/L)	0.024(-0.020, 0.067)	0.067	0.285
BUN (mg/dL)	-0.012(-0.264, 0.239)	-0.006	0.923
Urea (mg/dL)	0.006(-0.124, 0.137)	0.006	0.925
Serum creatinine (mg/dL)	-3.296(-7.820, 1.228)	-0.090	0.153
Albumin (g/dL)	-0.064(-0.277, 0.149)	-0.037	0.555
Globulin (g/dL)	0.422(-2.366, 3.211)	0.019	0.766
Total protein (g/dL)	-0.062(-0.275, 0.152)	-0.036	0.570
Gender, n (%)	-0.693(-3.172, 1.786)	-0.035	0.582
Smoking status, n (%)	1.710(-0.830, 4.250)	0.083	0.186
Alcohol consumption, n (%)	0.304(-0.946, 1.554)	0.030	0.633
Exercise activity, n (%)	-0.259(-2.699, 2.180)	-0.013	0.834
CKD stage, n (%)	-0.936(-2.942, 1.070)	-0.058	0.359
USG fatty liver, n (%)	0.121(-1.639, 1.881)	0.009	0.892
Diabetic status, n (%)	0.791(-0.199, 1.781)	0.099	0.117

The multivariate regression analysis for METS-IR in Table 3 showed significant positive associations with fasting blood sugar (B=0.489, p<0.001) and fasting TG (B=0.022, p=0.003). HDL-c (B=-0.168, p=0.005) and albumin (B=-0.168, p=0.005) showed significant negative associations with METS-IR, indicating that higher HDL-c and albumin levels are beneficial in reducing insulin resistance. GGT (B=0.059, p=0.022) was also significantly associated with METS-IR, suggesting the role of liver function in insulin resistance. Other variables such as age, height, weight, WC, BMI, PPBS, HbA1c, eGFR, total cholesterol, LDL-c, AST, ALT, BUN, urea, serum creatinine, globulin, total protein, gender, smoking status, alcohol consumption, exercise activity, CKD stage, and USG fatty liver did not show significant associations with METS-IR in this model.

**Table 3:** Results of multivariate analysis of METS-IR unadjusted model in the whole population BMI, body mass index; FBS, fasting plasma glucose; PPBS, post-prandial plasma glucose; METS-IR, metabolic score for insulin resistance; eGFR, estimated glomerular filtration rate; TG, triglycerides; LDL-C, low-density lipoprotein cholesterol; HDL-C, high‐density lipoprotein cholesterol; AST, aspartate aminotransferase; ALT, alanine aminotransferase; GGT, gamma-glutamyl transpeptidase; BUN, blood urea nitrogen; CKD, chronic kidney disease; USG, ultrasonography; B, unstandardized coefficient; CI, confidence interval; β, standardized coefficient.

Variable	B (95%CI for B)	β	P-value
Constant	-3.705(-104.311, 96.901)	-	0.942
Age	0.069(-0.035, 0.173)	0.107	0.195
Height (cm)	0.003(-0.587, 0.592)	0.002	0.993
Weight (kg)	-0.012(-0.672, 0.647)	-0.016	0.970
Waist circumference (cm)	-0.029(-0.099, 0.042)	-0.054	0.429
BMI (kg/m^2^)	1.664(-0.088, 3.417)	0.720	0.062
FBS (mg/dL)	0.049(0.020, 0.078)	0.318	0.001
PPBS (mg/dL)	-0.017(-0.040, 0.005)	-0.176	0.128
HbA1c (%)	0.065(-0.787, 0.917)	0.014	0.881
METS-IR	Dependent variable	-	-
eGFR (mL/min/1.73 m^2^)	0.031(-0.097, 0.158)	0.069	0.637
Fasting TG (mg/dL)	0.022(0.008, 0.037)	0.168	0.003
Total cholesterol (mg/dL)	-0.009(-0.044, 0.027)	-0.038	0.635
LDL-c (mg/dL)	-0.017(-0.057, 0.023)	-0.066	0.397
HDL-c (mg/dL)	-0.168(-0.284, -0.052)	-0.171	0.005
AST (IU/L)	-0.002(-0.036, 0.033)	-0.008	0.923
ALT (IU/L)	-0.023(-0.104. 0.058)	-0.056	0.576
GGT (IU/L)	0.059(0.009, 0.110)	0.169	0.022
BUN (mg/dL)	0.121(-0.330, 0.572)	0.060	0.597
Urea (mg/dL)	-0.041(-0.290, 0.208)	-0.039	0.745
Serum creatinine (mg/dL)	-1.426(-11.375, 8.523)	-0.039	0.778
Albumin (g/dL)	variable excluded; tolerance = 0		
Globulin (g/dL)	0.482(-1.786, 2.750)	0.021	0.676
Total protein (g/dL)	0.024(-0.145, 0.193)	0.014	0.782
Gender, n (%)	-0.099(-3.365, 3.166)	-0.005	0.952
Smoking status, n (%)	0.299(-2.942, 3.539)	0.015	0.856
Alcohol consumption, n (%)	0.192(-1.302, 1.687)	0.019	0.800
Exercise activity, n (%)	0.486(-1.379, 2.350)	0.025	0.608
CKD stage, n (%)	1.897(-1.382, 5.176)	0.117	0.256
USG fatty liver, n (%)	-1.035(-2.452, 0.383)	-0.073	0.152
Diabetic status, n (%)	-0.745(-1.724, 0.234)	-0.093	0.135

The correlation analysis between METS-IR and eGFR in Table 4 indicated a weak and nonsignificant association between insulin resistance and renal function in the studied population. Pearson's correlation coefficient was 0.020 (p=0.756), and Spearman's rho was 0.021 (p=0.739), indicating minimal positive relationships that were not statistically significant.

**Table 4:** Combined correlation analysis between METS-IR and eGFR METS-IR, metabolic score for insulin resistance; eGFR, estimated glomerular filtration rate.

Correlation Method	Correlation Coefficient	Significance (Two-Tailed)
Pearson correlation	0.020	0.756
Spearman’s rho	0.021	0.739

**Figure 1:**
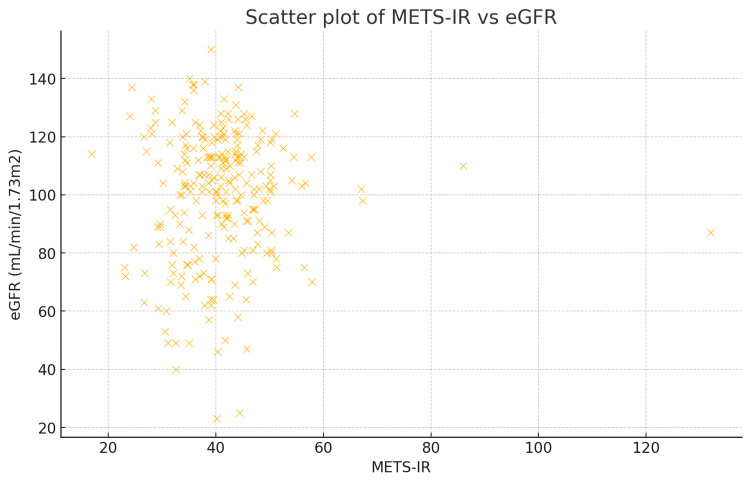
Scatter plot of METS-IR and eGFR METS-IR, metabolic score for insulin resistance; eGFR, estimated glomerular filtration rate.

Scatter plot analysis (Figure 1) showed a slight negative trend between METS-IR and eGFR, indicating that higher METS-IR values are associated with lower eGFR in this study population.

**Figure 2:**
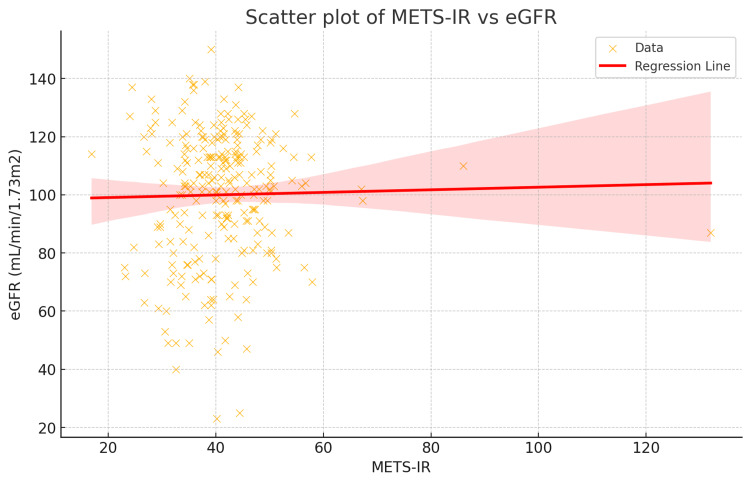
Scatter plot of METS-IR and eGFR with regression line and shaded confidence interval METS-IR, metabolic score for insulin resistance; eGFR, estimated glomerular filtration rate.

The scatter plot of METS-IR vs. eGFR and the regression line indicated that higher levels of insulin resistance are associated with poorer kidney function but do not significantly depict the negative association between METS-IR score and eGFR. The plot includes a regression line with a shaded confidence interval, as shown in Figure 2. The regression line is relatively flat, indicating a weak linear relationship between METS-IR and eGFR in this study population. The wide confidence interval suggests a high degree of variability in this data.

## Discussion

METS-IR, a newly developed score to measure IR, is not dependent on fasting insulin levels but on lipid profile, blood glucose, and BMI, which are easily accessible even in primary healthcare centers [11,12]. HEC is considered the prime way to measure IR, but it is invasive and does not hold well for a broader population [11,13]. However, HOMA-IR serves as the most approved and reliable indicator of IR that is widely used in clinical research [12,14]. However, because of its cost, especially in underdeveloped areas, its usage in routine clinical practice is limited, and a need for an alternative has arisen [12,15]. Our study assesses the utility of the METS-IR score in depicting the metabolic risk associated with this cohort of the South Indian population.

The analysis of baseline characteristics across quartiles of METS-IR highlights the complex interplay among metabolic health and clinical parameters. Notably, higher METS-IR is significantly associated with increased weight, WC, and BMI, highlighting the role of adiposity in metabolic risk. Elevated blood glucose levels and HbA1c across higher METS-IR quartiles emphasize the nexus between insulin resistance and glycaemic control. Additionally, dyslipidemia, as evidenced by rising fasting TG and LDL-c levels, further elucidates the metabolic derangements accompanying higher METS-IR. The significant variation in CKD stages across METS-IR quartiles suggests a concerning association between renal function and metabolic health. Advocating an integrated approach to managing metabolic health, addressing not only glucose control but also weight management and lifestyle modifications, lipid profile optimization, and renal health preservation, is pivotal.

Univariate regression links METS-IR with age, weight, WC, BMI, FBS, and TG, emphasizing adiposity and glycaemic control. Multivariate analysis identifies FBS and TG as strong positive predictors. HDL-c shows a significant negative association, indicating benefits in reducing insulin resistance. GGT's positive association focuses attention on the importance of liver function. Elevated METS-IR scores are closely linked to increased adiposity, as indicated by higher weight, WC, and BMI, all of which are well-documented risk factors for CVD. Dyslipidemia, characterized by higher fasting TG and LDL-c levels, further underscores the connection between METS-IR and cardiovascular risk [16]. These findings suggest that individuals with higher METS-IR scores are at a greater risk of developing cardiovascular complications, highlighting the importance of using METS-IR as a predictive tool in clinical practice.

Some studies failed to find a significant association between IR and eGFR. Johns et al. noted no connection between IR and eGFR, even though patients with metabolic disorders had an increased risk of developing CKD in the future and a lower eGFR when compared with the control population [17]. In another study conducted on subjects ≥20 years old without established diabetes who had routine health checkups, there was no significant association between HOMA-IR and eGFR. ANOVA-adjusted HOMA-IR values in lesser eGFR groups were not predominantly higher. It was concluded that there were no remarkable variations in HOMA-IR values concerning components of metabolic syndrome. Hence, they concluded that decreased renal function in relation to IR has uncoupled significance [18]. Similarly, our study shows a weak significance between the METS-IR with eGFR and the CKD stages in those individuals who underwent a master health checkup. Other studies that have made similar conclusions regarding ethnic and racial groups have been documented [19-21]. This study was conducted without deliberate exclusion of people with CKD and diabetes, which is factual. METS-IR is a new IR index alternative to HOMA-IR that is even better at measuring insulin resistance [11,22].

The correlation analysis between METS-IR and eGFR indicates a weak and nonsignificant linear association. However, higher METS-IR scores had a significant positive correlation with age and serum creatinine independently, but collectively, poor significance was observed with eGFR and different CKD stages, thus suggesting the role of other probable confounding covariates would play a critical role in maintaining renal function in this cohort. Our study's findings are more consistent with that of Shi et al. [23]. Higher scores of METS-IR were associated with a mild reduction in eGFR and thereby proposed to be accompanied by a high risk of hastened decline in eGFR. Therefore, it was concluded that METS-IR was a suitable indicator to identify the risk of renal dysfunction in its early stages [24].

CKD and metabolic syndrome are closely associated; the kidney is the earliest sensitive target organ [25]. Multiple studies have shown substantial results that link chronic inflammation and insulin resistance to metabolic syndrome, which has a role in defining abnormalities in glucose and fat metabolism [26-28]. Similarly, our study shows no significant correlation between the METS-IR with eGFR and the progression of CKD among individuals who underwent master health checkups, eliminating those who had established diabetes. These findings emphasize the need for a comprehensive approach to managing metabolic and renal health, focusing on aging, glycemic control, lipid metabolism, and renal function monitoring.

Strengths and limitations

The score is easily accessible and highly applicable, even in resource-limited primary healthcare settings. It can be implemented during regular master health checkups and used for the primordial detection of risks associated with metabolic syndrome.

This retrospective single-center study may not be generalizable to other populations. Data inconsistencies and missing information from electronic medical records could affect results. Measurement variability and the lack of longitudinal follow-up limit causal inferences. Including heterogeneous diabetic statuses introduces variability. The reliance on the relatively new METS-IR tool, without comprehensive validation in the South Indian population, adds uncertainty. Additionally, the study did not assess inflammatory markers, which are relevant to metabolic and renal health.

## Conclusions

According to this study, METS-IR had a strong positive linear relationship with cardiovascular health rather than eGFR. However, poorer renal function is linked with high METS-IR scores. By identifying the at-risk individuals attending master health checkups using the METS-IR score as a screening tool, healthcare providers can implement targeted primordial and primary interventions, thereby focusing on weight management, glycemic control, and lipid and renal profile optimization, further mitigating the risk of developing metabolic syndrome. Hence, it plays a potential role in guiding preventive strategies in a tertiary care setting.

## References

[1] Medeiros CC, Ramos AT, Cardoso MA, França IS, Cardoso Ada S, Gonzaga NC (2011). Insulin resistance and its association with metabolic syndrome components. Arq Bras Cardiol.

[2] Gluvic Z, Zaric B, Resanovic I, Obradovic M, Mitrovic A, Radak D, Isenovic ER (2017). Link between metabolic syndrome and insulin resistance. Curr Vasc Pharmacol.

[3] Kilpatrick ES, Rigby AS, Atkin SL (2007). Insulin resistance, the metabolic syndrome, and complication risk in type 1 diabetes: "double diabetes" in the Diabetes Control and Complications Trial. Diabetes Care.

[4] Moller DE, Flier JS (1991). Insulin resistance—mechanisms, syndromes, and implications. N Engl J Med.

[5] González-González JG, Violante-Cumpa JR, Zambrano-Lucio M (2022). HOMA-IR as a predictor of health outcomes in patients with metabolic risk factors: a systematic review and meta-analysis. High Blood Press Cardiovasc Prev.

[6] Cheng H, Jia Z, Li YT (2024). Metabolic score for insulin resistance and new-onset type 2 diabetes in a middle-aged and older adult population: nationwide prospective cohort study and implications for primary care. JMIR Public Health Surveill.

[7] Charles K, Lewis MJ, Montgomery E, Reid M (2024). The 2021 chronic kidney disease epidemiology collaboration race-free estimated glomerular filtration rate equations in kidney disease: leading the way in ending disparities. Health Equity.

[8] Chen RY, Shi J (2024). Evaluation of the CKD-EPI 2021 creatinine equation using laboratory data: considerations for practice changes among clinical laboratories in British Columbia, Canada. Clin Biochem.

[9] Veltkamp DM, Rookmaaker MB, Verhaar M, van Solinge WW, Haitjema S, Vernooij RW (2024). #2514 Clinical impact of the CKD-EPI 2021 versus the CKD-EPI 2012 formula on GFR estimation and CKD prevalence: results from a Dutch routine-care cohort. Nephrol Dial Transplant.

[10] Saadeh S, Younossi ZM, Remer EM (2002). The utility of radiological imaging in nonalcoholic fatty liver disease. Gastroenterology.

[11] Bello-Chavolla OY, Almeda-Valdes P, Gomez-Velasco D (2018). METS-IR, a novel score to evaluate insulin sensitivity, is predictive of visceral adiposity and incident type 2 diabetes. Eur J Endocrinol.

[12] Han KY, Gu J, Wang Z (2022). Association between METS-IR and prehypertension or hypertension among normoglycemia subjects in Japan: a retrospective study. Front Endocrinol (Lausanne).

[13] DeFronzo RA, Tobin JD, Andres R (1979). Glucose clamp technique: a method for quantifying insulin secretion and resistance. Am J Physiol.

[14] Borai A, Livingstone C, Kaddam I, Ferns G (2011). Selection of the appropriate method for the assessment of insulin resistance. BMC Med Res Methodol.

[15] Manley SE, Stratton IM, Clark PM, Luzio SD (2007). Comparison of 11 human insulin assays: implications for clinical investigation and research. Clin Chem.

[16] Qian T, Sheng X, Shen P, Fang Y, Deng Y, Zou G (2023). Mets-IR as a predictor of cardiovascular events in the middle-aged and elderly population and mediator role of blood lipids. Front Endocrinol (Lausanne).

[17] Johns BR, Pao AC, Kim SH (2012). Metabolic syndrome, insulin resistance and kidney function in non-diabetic individuals. Nephrol Dial Transplant.

[18] Park JH, Oh SW, Ahn SY (2013). Decreased estimated glomerular filtration rate is not directly related to increased insulin resistance. Diabetes Res Clin Pract.

[19] Yoon J, Heo SJ, Lee JH, Kwon YJ, Lee JE (2023). Comparison of METS-IR and HOMA-IR for predicting new-onset CKD in middle-aged and older adults. Diabetol Metab Syndr.

[20] Cai Q, Wang X, Ye J, Zhuo L, Song H, Liu C, Zhuo L (2016). Metabolic syndrome does not always play a critical role in decreased GFR. Ren Fail.

[21] Li Y, Xie D, Qin X (2015). Metabolic syndrome, but not insulin resistance, is associated with an increased risk of renal function decline. Clin Nutr.

[22] Ko J, Skudder-Hill L, Tarrant C, Kimita W, Bharmal SH, Petrov MS (2021). Intra-pancreatic fat deposition as a modifier of the relationship between habitual dietary fat intake and insulin resistance. Clin Nutr.

[23] Shi W, Liu S, Jing L, Tian Y, Xing L (2019). Estimate of reduced glomerular filtration rate by triglyceride-glucose index: insights from a general Chinese population. Postgrad Med.

[24] Wang P, Li Q, Guo X (2021). Usefulness of metabolic score for insulin resistance index in estimating the risk of mildly reduced estimate glomerular filtration rate: a cross-sectional study of rural population in China. BMJ Open.

[25] Prasad GV (2014). Metabolic syndrome and chronic kidney disease: current status and future directions. World J Nephrol.

[26] Turcotte LP, Fisher JS (2008). Skeletal muscle insulin resistance: roles of fatty acid metabolism and exercise. Phys Ther.

[27] Semenkovich CF (2006). Insulin resistance and atherosclerosis. J Clin Invest.

[28] Bailey JL, Zheng B, Hu Z, Price SR, Mitch WE (2006). Chronic kidney disease causes defects in signaling through the insulin receptor substrate/phosphatidylinositol 3-kinase/Akt pathway: implications for muscle atrophy. J Am Soc Nephrol.

